# The Association of Cancer Physicians Breast Cancer Forum: Implications of Calman–Hine for breast cancer services

**DOI:** 10.1054/bjoc.2000.1235

**Published:** 2000-06-02

**Authors:** R C F Leonard

**Affiliations:** Department of Clinical Oncology, Western General Hospital, Edinburgh, EH4 2XU, UK


*British Journal of Cancer* (2000) **83**(1), 135–140
© 2000 Cancer Research Campaign
doi: 10.1054/ bjoc.2000.1235, available online at http://www.idealibrary.com on IDEAL®

Meeting report

### The Association of Cancer Physicians Breast Cancer Forum: Implications of Calman–Hine for breast cancer services

**RCF Leonard**

Department of Clinical Oncology, Western General Hospital, Edinburgh EH4 2XU, UK

### 12 March 1999, Royal College of Physicians, London, organized by Darwin Medical Communications Ltd, Abingdon, Oxfordshire, UK.

The meeting was organized in the light of the impetus from health authorities to push ahead with implementation of the Calman–Hine Report in breast cancer services. This is an appropriate time to review progress in implementation so far and to assess problems.

### Background to Calman–Hine and guidelines for breast cancer management – *Robert Haward (University of Leeds, UK)*

The rationale for Calman–Hine (C–H) (Department of Health, 1995) and its equivalents in Scotland and Northern Ireland, was to eliminate significant variations in cancer management within the UK – variations which have proved very persistent. The nature and extent of this variation can be inferred from cancer registry data, from audits, and from other observational studies, rather than from randomized trial evidence. There has been a public perception of significant geographical differences in access to cancer services, in diagnostic and treatment approaches, and in communications. This contributed to the political pressure for something to be done.

The Expert Advisory Group on Cancer (EAGC) which produced C–H reviewed the evidence in a number of key areas. They concluded that there was a real problem, that there were strong grounds for systematic specialization in the delivery of cancer care, and that multidisciplinary teams were an important factor in improving the service. The evidence for the effectiveness of a network system of care – the unit-centre model, linked to primary care – is rather weaker, but there is evidence that collaboration involving people in different institutions can lead to better and more consistent care.

The aim of the strategy implicit in C–H's recommendations was the improvement of local delivery of services, using documented, audited and monitored procedures, and ensuring proper linkage between the different aspects of delivery. C–H saw cancer services commissioning as a key potential influence on the implementation of the necessary changes. Other necessary elements to support implementation were guidelines and the systematic recording of agreed data. Guidelines can be either clinical, like those from BASO (British Association of Surgical Oncology, 1995) or about service delivery – which led to the production of the national COG (Cancer Guidance Sub-Group of the Clinical Outcomes Group) guidance. Initiatives to encourage the use of systems for collection of data are now beginning to come through e.g. the Royal College of Pathologists' minimum reporting standards, and minimum datasets and associated databases defined by various national associations such as BASO.

The first national COG guidance related to services for the management of breast cancer (COG, 1996). A great deal of activity has followed in relation to setting up the right structures for breast services in cancer units and centres, although the precise process has varied markedly across the country. The most significant recommendations in both the clinical guidelines and national guidance were for the constitution of specialist breast teams in cancer units and centres. Much effort has been applied throughout the UK to constitute such teams and to get them to work effectively, with all breast patients referred to them. The concept of triple assessment has also been widely embraced. Less consistent progress has been made in areas such as communication and monitoring outcomes.

The great majority of symptomatic breast patients are now seeing designated surgeons. Most surgeons are beginning to work in teams with appropriate colleagues, despite practical restrictions. The quality of team-working within these specialist teams varies considerably, however. The variations can partly be explained by logistical difficulties and are sometimes a result of the shortage of trained people to fill vacant posts. There are also problems in establishing meaningful communication between specialist teams in hospitals and primary care. More rigorous quality assurance and more peer-review visiting would be beneficial.

Considerable change and a lot of progress has been made over the last decade since breast screening was generally introduced. It has been followed by C–H, a framework for all cancer services, and by the detailed COG guidance. While this broke new ground in seeking to standardize the way breast cancer services are delivered across the NHS, there is still a long way to go.

### Review of radiology/screening – Robin Wilson (City Hospital, Nottingham, UK)

The introduction of the National Breast Screening Programme (NHSBSP) arguably provided the driving force behind all the measures under discussion at the meeting. Breast cancer care is viewed as the model for care in other kinds of cancer. The

*Received 20/09/99*
*Accepted 13/03/00*

radiologist is a core member of the multidisciplinary breast team and is fundamental to accurate diagnosis (Teh et al, 1998; Blamey, 1998), preoperative staging of cancer, surveillance after treatment, and restaging for recurrent or metastatic disease.

The NHSBSP has demonstrated the value to patients of integrating imaging into the primary diagnostic process. The radiologist needs to attend new-referral clinics alongside the surgeon, providing instant reporting of mammograms and carrying out ultrasound scans and image-guided biopsy. In the assessment of patients with breast cancer, the mammogram and ultrasound features are fundamental in determining the disease extent and whether conservation surgery is feasible. During initial diagnosis and treatment, the radiologist must also participate in the multidisciplinary clinical meetings along with the surgeon, pathologist and oncologist to ensure that the most appropriate management decisions are made for each patient. Some 40 standards have been published within the NHSBSP, and a few more are in the pipeline. The Royal College of Radiologists, Royal College of Radiologists Breast Group, BASO, and the British Breast Group have all, over the last 5 years, produced their own guidelines.

Radiologists involved in the breast-screening programme have all the skills required for symptomatic breast imaging, and it is only sensible that the same radiologists should also support symptomatic breast radiology. However, the extension of the radiologist's role in symptomatic breast care is considerably increasing the demand for specialist radiology time. This is on top of increasing demands within the NHSBSP and compounded by changes in practice in breast diagnosis, with radiologists now carrying out needle biopsies of breast abnormalities, under image guidance, even for palpable lesions. Radiology is now the rate-limiting step that determines how fast patients are seen in the clinic. Features of lumps can now be distinguished which go beyond the solid/cystic differentiation to which ultrasound has traditionally been restricted. Tumours can be diagnosed when they are much smaller than was previously possible. High-frequency ultrasound is now essential in breast cancer diagnosis. Doppler allows assessment of the vascularity of the tumour. Doppler contrast, and harmonics, allow still more sophisticated investigations. As regards biopsy techniques, fine-needle aspiration (FNA) results are disappointing. This year, less than half of screening programmes have reached the target of 70% of cancers diagnosed preoperatively (Blanks R, personal communication). Core biopsy is supplanting it. Using core biopsy under image guidance, it should be possible to diagnose up to 95% of cancers preoperatively. Ultrasound-guided vacuum biopsy is expensive but highly accurate (Parker et al, 1996).

In many parts of the UK there is a shortage of radiologists specializing in breast imaging. The pressures of the NHSBSP quality-assurance programme have reduced the enthusiasm of radiologists in training to consider specialization in breast imaging – radiology is currently the only breast care specialty whose practitioners require to be individually peer reviewed. Twenty percent of breast posts advertised last year are still vacant. Breast radiologists are bearing the brunt of the heightened public awareness of, and tendency to pursue, litigation in relation to cancer screening programmes. According to a report published in 1998, 28% of respondents were, at the time of survey, involved in some kind of medico-legal action citing delayed diagnosis. Also, 94% were experiencing an increased workload, and 83% believed that standards had dropped as a result. Morale was found to be low, and 42% were seriously considering leaving (Field, 1998). To help alleviate the manpower shortage, a working party set up by the Chief Medical Officer has proposed that non-radiographers be deployed to take films, while radiographers will read them. Despite this, there will need to be a significant expansion in numbers of breast radiologists, and considerable investment in equipment in the clinic, if the expertise required for quality breast cancer diagnosis and treatment is to be provided.

### Role of clinical oncologist in guideline development – *Ian Kunkler (Western General Hospital, Edinburgh, UK)*

The clinical oncologist is involved in the delivery of radiotherapy and cytotoxic therapy for breast cancer and forms part of a multidisciplinary team including breast surgeons, medical oncologists, radiologists, pathologists, general practitioners, cancer nurses and radiographers. It is envisaged that national guidelines such as those drawn up by the Scottish Intercollegiate Guidelines Network (SIGN, 1998) and the Royal College of Radiologists Clinical Oncology Information Network (COIN, in preparation), should form the basis for the development of locally based guidelines in cancer centres and cancer units. Most clinical oncologists undertake the non-surgical management of breast cancer in both cancer units and cancer centres. They are therefore well placed to ensure that the local guidelines in cancer centres and cancer units, developed from such national guidelines, are consistent with one another.

In developing guidelines the clinical oncologist may contribute to defining best practice in staging, selection of appropriate adjuvant radiotherapy and systemic therapy following breast-conserving therapy or mastectomy, the management of locally advanced, recurrent and metastatic disease, and policies of post-treatment surveillance.

The guiding principles in writing guidelines are: (a) the quality of the evidence; (b) an interactive process by which draft guidelines are revised and improved by peer review; (c) avoidance of dogma where there is insufficient evidence to support firm recommendations; (d) acceptability to the professional, and (e) clarity of expression. The highest level of evidence (level 1a) is based on a meta-analysis of randomized controlled trials. The lowest level (level 4) is based on expert opinion.

Assessing the quality of the evidence is not easy. For example, the apparent lack of any improvement in survival from postoperative radiotherapy in an overview of randomized trials (EBCTCG, 1995) may be at odds with a large individual trial (Overgaard et al, 1997) that does show a survival advantage.

Efforts continue to improve radiotherapy techniques, and this of course has implications for local control and minimization of morbidity. One problem is the uneven contour of the breast, which gives rise to local hot-spots in radiotherapy, which may result in local morbidity. The larger the breast, the greater its heterogeneity (Neal et al, 1995) and the more likely a poor cosmetic result because of imbalances in dose. New techniques for 3D planning of the breast involve rapid acquisition of CT slices throughout the whole substance of the breast: the path length of each slice can be computed rapidly, and sophisticated planning algorithms can be used to optimize the distribution of radiotherapy over the breast (Carruthers et al, 1999). The equipment necessary is expensive, however, and a cost–benefit judgement has to be made in formulating relevant guidelines.

© 2000 Cancer Research Campaign

Regarding breast conservation, SIGN (1998) guidelines state that radiotherapy should conventionally be given after wide excision or quadrantectomy. Data from the Scottish Conservation Trial on women under 69 years deemed suitable for conservation therapy, who received appropriate systemic therapy based on ER status, and who were then randomly assigned to radiotherapy or no radiotherapy, showed a significant advantage in ipsilateral recurrences for the radiotherapy group (Forrest et al, 1996). Over the whole study there was a 4 fold reduction in risk of local recurrence for patients receiving radiotherapy. Disadvantages of breast irradiation include acute and late toxicity and hospitalization for frail patients or those living too far from the cancer centre for outpatient treatment.

The issue of treatment of the axilla continues to arouse controversy. Advice from SIGN (Chetty et al, 1998) is that, after axillary sampling, the axilla should only be irradiated if patients are node-positive or if they have been inadequately sampled. (However, in the speaker's view, the axilla should not be irradiated unless adequately sampled, because of the risks of late morbidity.) A recent trial at Edinburgh examined the morbidity of axillary surgery – sample or clearance – after wide excision. Patients were randomized to axillary node sample or level 3 clearance. Those sampled and found node-positive received radiotherapy; those node-negative or who received clearance, did not. The irradiated group experienced greatest morbidity in terms of shoulder power, followed by the clearance group. The clearance group fared worst in terms of lymphoedema. There was no difference in survival or axillary recurrence.

Also controversial is the issue of post-mastectomy radiotherapy. The recent SIGN (1998) guidelines, influenced by trials from Denmark and Canada, suggest that adjuvant post-mastectomy radiotherapy should be given to patients at high risk of local occurrence, taking into account nodal positivity, lymphovascular invasion, grade and size of tumour (although it is not prescriptive about how these factors should be summated). On the basis of a 1987 overview of postoperative radiotherapy trials, which showed increased mortality for patients undergoing post-mastectomy irradiation, many surgeons stopped referring patients for post-operative radiotherapy. Clinical oncologists were aware that, within the overview, there was a wide variety of treatment techniques used and that, in the early part of the period covered by the review, the orthovoltage radiation used gave a high dose to the heart. With modern megavoltage radiation, dosage to the heart is relatively small. A re-analysis showed that there was a trend to a small increase in survival in the irradiated group. A more recent, large ($n$ = 1708) trial (Overgaard et al, 1997) in high-risk premenopausal women randomized post-mastectomy to adjuvant chemotherapy alone or in conjunction with radiotherapy, and found a survival advantage for the irradiated group (48% vs 34% at 10 years). The clinical oncology community is currently assessing the implications for practice.

### The model of joint care: surgeons and oncologists – *Robert Mansel (University Hospital of Wales, Cardiff, UK)*

Change arising from C–H has arguably been greater for the surgeon than for the oncologist. As has been stated earlier, the main driving force behind C–H has been the Breast Screening Programme. In the USA, voluntary groups have been successful in diverting substantial amounts of money into health care from other budgets, e.g. defence; not so in the UK. BASO has had to struggle against opposition from general surgical groups to introduce breast specialism. (Rescheduling of clinics has been achieved at Cardiff only after $2\frac{1}{2}$ years of negotiation.)

Practical problems, e.g. regarding the availability of oncologists, may force departures from the ideal situation envisaged by C–H in which, following triple assessment on a same-day or rapid-access basis, the patient has a care plan drawn up at a multidisciplinary meeting attended by oncologist and surgeons. Also, few clinics draw up treatment plans in a readily auditable form.

As regards documentation, BASO has done an enormous amount of work: the second version of the breast database has just been published, the second edition of the guidelines has recently been published, and the second edition of the primary care referral guidelines consonant with the new 2-week rule will be published very soon. The last is a very important instrument for the future success of C–H implementation.

At Cardiff, a weekly clinic is held with all four surgeons together in the same clinic. (This is useful for training, since there is always a consultant present.) For rapid communication, there is a dedicated fax line for GPs to fax proformas. Patients are automatically invited for mammograms after they reach the age of 35. All investigations are performed on a one-stop basis, but not results. All results are fed into a weekly multidisciplinary meeting prior to seeing the patient to give the diagnostic results. Patients are seen and get results within 3 days.

Recording the decisions made at the multidisciplinary meeting allows comparison with existing guidelines and also comparison with an ideal management for that patient's subgroup, using overview data. Studies looking at specialist care vs non-specialist care in Scotland suggest that not only is the quality of the staging better but there is lower local recurrence (by a factor of three) and improved mortality in those patients who are looked after in a specialist setting involving multidisciplinary working.

Outcome in terms of cosmesis needs more attention. Current data systems don't acquire this. Breast reconstruction still shows marked variation in settings, outcomes and evaluation across different locations. The BASO database in the symptomatic area allows ready assessment of quality-of-work by individual surgeons and can be a valuable indicator of training needs.

An important caveat needs to be registered, however. The incidence of cancer among patients referred to breast clinics used to be about 1 in 10; in a recent survey at the Cardiff breast clinic, out of 2333 referrals, there were 147 symptomatic carcinomas: 6%. This represents one of the pressures imposed on the quality of care which can be offered to the woman who does have cancer. The referral guidelines take into account factors such as age, for example. The second edition also includes a short statement about family history. Comparing GP letters with the referral guidelines showed that, if the guidelines had been followed, all of the symptomatic cancers would have been referred, but 29% of the total population would not have needed referral. Most patients do not need triple assessment; they need counselling, advice, and to talk about family history. There is a major issue here which poses a threat, to C–H organization in the breast cancer world, since the clinics will increasingly become populated by the 'worried well'.

The issue of follow-up is highly contentious. COG showed that follow-up is unnecessary, but this view is robustly opposed by many surgeons. Are we providing a quality service? Data show

© 2000 Cancer Research Campaign 

that nearly all local recurrences are detected by the woman herself. Why not an alternative model? One could have shared primary care and breast clinic care, which might reduce the hospital load, leaving more time for new patients, maintain the database by better IT from primary care to breast clinic, and have a mammographic surveillance for follow-up.

Overall conclusions are that multidisciplinary working with minimum standards and national guidelines should lead to better outcomes. But we should remember that future improvements in mortality are likely to be modest.

### Chemotherapy – *Mary O'Brien (Royal Marsden Hospital, London, UK)*

About a quarter of spending on anticancer drugs is for breast cancer. The medical oncology speciality, originally largely research-based, has developed into one providing a broad range of opinion on cancer treatment, albeit with an emphasis on drug treatment. The specialty's evidence-based orientation has contributed to the accumulation of a body of mature studies, many with 10–15 years follow-up.

Recent developments in chemotherapy for both early and advanced breast cancers have important implications for the costing and delivery of services as outlined in C–H.

A recent overview of over 50 adjuvant chemotherapy trials involving 30 000 women established new criteria for significant survival benefit (EBCTCG 1998*a*). The first key finding was that combination chemotherapy produced highly significant proportional reductions in the odds of death both for women under the age of 50 years and for women aged 50–69 years. Reductions in recurrence emerged chiefly during the first 5 years of follow-up, whereas survival differences grew throughout the first 10 years. An important finding was that the proportional reductions in risk were similar for women with both node-negative and node-positive disease, although the absolute reduction was greater in node-positive women with a higher absolute risk. The age-specific benefits were largely irrespective of menopausal status at presentation, oestrogen receptor status of the primary tumour and of whether adjuvant tamoxifen had been given or not. There was also the suggestion that anthracycline-containing regimens have significantly greater benefits on recurrence and survival than more traditional CMF schedules. Adjuvant trials with new agents including the taxanes are currently underway, and preliminary data from one such trial suggests a small but statistically significant further survival gain for the use of paclitaxel following standard adriamycin/cyclophosphamide chemotherapy.

These findings suggest that more women are likely to gain from adjuvant chemotherapy than was previously realized, with consequent implications for the resourcing and delivery of chemotherapy services.

Preoperative chemotherapy is an important new approach to the treatment of early breast cancer, using the primary tumour as an in vivo measure of responsiveness to therapy. Current evidence from randomized trials suggests that survival is very similar whether such treatment is given before or after surgery, but the need for mastectomy is reduced when chemotherapy is used preoperatively. This approach requires the closest possible cooperation between the oncologist and the surgeon working together with patients in multidisciplinary clinics from the time of first-diagnosis. This in turn has implications for the organization and staffing of breast clinics.

Several new drugs, in particular paclitaxel, docetaxel and vinorelbine, have been shown to be very active in the treatment of metastatic breast carcinoma. For the first time, data from randomized trials using these drugs suggest a survival benefit over some conventional schedules in patients with metastatic disease. Evidence also exists indicating symptom-relief and quality-of-life benefits. The key issue here concerns cost: some of these new agents are much more expensive than conventional therapy. Currently, 'rationing' of new therapies is determined by individual authorities, leading to so-called 'postal code prescribing'. A national policy for determining cost-effectiveness of new chemotherapies in patients with metastatic breast cancer is urgently required.

### Endocrine therapy – *Anthony Howell (Christie Hospital, Manchester, UK)*

Endocrine treatment is the preferred first-line therapy for advanced breast cancer because of its good tolerability. In addition, up to half of patients will respond again to endocrine therapy second-line, and promising results have been obtained with endocrine agents in third- and fourth-line treatment. Thus the aim of endocrine therapy should be to achieve as great a response as possible first-line. At present, tamoxifen is the drug of choice as first-line endocrine therapy for metastatic breast cancer with no or minimal symptoms in premenopausal or postmenopausal women. New drugs such as second-and third-generation aromatase inhibitors and new anti-oestrogens have recently been introduced in the clinic or are in late development.

Apparent differences in the effectiveness of adjuvant therapies according to age has led to controversy concerning the optimum treatment for women with breast cancer above and below 50 years of age. In all cases the treatment of choice for ER-positive patients is tamoxifen. Ideally this should be given for a period of 5 years. An overview of randomized trials using adjuvant tamoxifen in women with early breast cancer confirmed that it is possible to improve the 10-year survival of women with ER-positive tumours, with proportional reductions in breast cancer recurrence and mortality (EBCTCG 1998*b*).

Thus the appropriate use of endocrine therapies in breast cancer depends on:

- Understanding the effect of endocrine therapies and the mechanisms of resistance associated with their use.
- Developing new agents with novel endocrine anti-tumour effects.
- Defining the best way to combine endocrine agents with cytotoxic or other endocrine agents.
- Identifying the long-term effects of endocrine agents in terms of disease control and prevention, as well as desirable and undesirable side-effects.

Endocrine therapy's uses in advanced disease and adjuvant therapy are well defined, and it must be offered. When endocrine therapy is no longer effective then chemotherapy should be considered with close consultation with the patient.

### Advantages and disadvantages of a multidisciplinary approach – *Lesley Fallowfield (University College Medical School, London, UK)*

There are few data on the advantages and disadvantages of a multidisciplinary team approach. Some of the putative advantages

*© 2000 Cancer Research Campaign*

are better outcomes from improved communication. However, the demonstrable lack of effective communication between specialists and departments can cause confusion for the patients about diagnosis, test results and future management plans. In the speaker's experience, even in good teams, members tend to be unaware of what other members are saying to patients. Too often, important information for the patient is omitted on the assumption that someone else must have relayed the relevant facts at appropriate times. This can be confusing and cause a loss of confidence in the team, provoking needless anxiety for the patients; it is also frustrating for clinicians who may have to spend extra time communicating quite basic information to an unprepared or misinformed patient.

A related issue concerns the benefits of rapid communication of results of investigations. Data emerging from the very few studies which have been conducted indicate that, particularly for one-stop clinics, while anxiety is indeed reduced for those who have benign disease, 1 day appears to be too rapid for those who have malignant disease confirmed. Women with malignant disease fared better where there was a 2–3 day delay between undergoing investigations and being informed of the results and treatment options. It may be that time to consider the implications of malignancy is beneficial to the patient's adaptation.

Quality and quantity of information need to be distinguished – more is not necessarily better. Theoretical improvements in communication provided by a multidisciplinary team may well be shown, but such approaches are alien to many health care professionals who have been educated within a hierarchical system. Hardly any time is devoted, at any level of training of medical personnel, to developing communication skills. Data from communication-skills training courses for senior doctors in cancer medicine and specialist chemotherapy nurses show that many find communication with colleagues one of the most stressful and unsatisfying aspects of their work. This may compound the stressors common to any organizational unit: role-uncertainty, role-ambiguity, role-conflict, role-overload, and so on. The vast literature accumulated by occupational psychologists could be brought to bear on these problems in the context of the multidisciplinary team. Unless old practices are abandoned, training provided and different patterns of communication established, then benefits to everyone in the system will not be realized.

### Primary care – *Ivan Cox (Laurie Pike Health Centre, Birmingham, UK)*

The reorganizations resulting from C–H are now impacting upon primary care, and GPs are coming to terms with the 'cancer pathway' model of care. The three areas in the management of breast cancer patients where this impact is being felt most are:

- early recognition, diagnosis and referral
- prescribing during remission
- follow-up.

'Fast tracking' and the '2-week rule' are having significant implications for GPs at both cultural and organizational levels. In particular, GPs are having to reconsider their own procedures in differentiating between potentially malignant and non-malignant breast lumps. This might lead to some GPs becoming 'de-skilled' in diagnosis, simply referring every suspicious patient. Further to this, they have to become familiar with the entry criteria and guidelines for admitting patients to the fast-track service. There is some evidence (both anecdotal or from small studies) that, even where local guidelines on how to get patients into fast-track systems exist, only about 30–40% of GPs use them, preferring instead to refer to a consultant familiar to them. By contrast, in places where use of the fast-track system has taken off, some GPs refer somewhat indiscriminately to the fast-track system. A small telephone survey by the speaker of six specialist clinics in Birmingham and Sandwell found that in some places the referral rate to fast-track clinics has risen by up to 100% in 2 years; concomitantly, in these units, the incidence of breast cancer detected among patients so referred has fallen from about 1 in 10–11 to about 1 in 18–24.

There are grave misgivings in general practice about the 2-week rule. Firstly, over its measurement. When does this period commence? From the first presentation or at the point when the referral is received by the specialists? There are some patients who do not want to be fast-tracked; they may want time to consider their position at each of the stages. The GP is also under pressure to pick up the fall-out from an increased number of worried-well patients resulting from fast-track clinics.

Further, with cash-limited prescribing budgets, some GPs are unhappy at having to pick up long-term prescriptions for the anti-oestrogens, particularly those in trials. The combined budgets to be given primary care groups may see an end to this bickering.

Though the debate over the value of follow-up itself is intensifying, patient pressure will undoubtedly force a significant amount of follow-up on the system – if only in the short term. The shift in resourcing to implement fast tracking is encouraging the specialist breast services to offload follow-up care. GPs are divided over the benefits of managing this in primary care. Again, resourcing is one of the issues of major concern.

However, one important outcome of the recent RCGP Cancer Care Workshops was the desire to work much more closely with specialists on all of these issues. The creation of primary care groups should encourage this further.

### REFERENCES

British Association of Surgical Oncology (1995) Guidelines for surgeons in the management of symptomatic breast disease in the United Kingdom. *Eur J Surg Oncol* **21** (Suppl A): 1–13. Revised (1998) *Eur J Surg Oncol* **24**(6): 464–476

Blamey RW (1998) The role of the radiologist in breast diagnosis: a surgeon's personal view. *Clin Radio* **53**: 393–395

Carruthers AJ, Redpath At and Kunkler IH (1999) The use of compensators to optimise the three dimensional dose distribution in radiotherapy of the intact breast. *Radiother Oncol* **50**: 291–300

Chetty U, Jack W, Dillon P, Tyler C and Prescott R (1998) Axillary surgery in patients with breast cancer being treated by breast conservation: a randomised trial of node sample or axillary clearance. First European Breast Cancer Conference. *Eur J Cancer* **34**(Suppl 5): S47

COG (Cancer Guidance Sub-group of the Clinical Outcomes Group) (1996) *Improving Outcomes in Breast Cancer: Guidance for Purchasers: The Manual* (1996*a*) and *The Research Evidence* (1996*b*). Department of Health: London

Department of Health (1995) *Policy Framework for Commissioning Cancer Services: a report by the Expert Advisory Group on Cancer to the Chief Medical Officers of England and Wales*. HMSO: London

EBCTCG (Early Breast Cancer Trialists' Collaborative Group) (1995) Effects of radiotherapy and surgery in early breast cancer – an overview of randomized trials. *N Engl J Med* **333**: 1444–1455

EBCTCG (Early Breast Cancer Trialists' Collaborative Group) (1998*a*) Polychemotherapy for early breast cancer: an overview of the randomised trials. *Lancet* **352**: 930–942

EBCTCG (Early Breast Cancer Trialists' Collaborative Group) (1998*b*) Tamoxifen for early breast cancer: an overview of the randomised trials. *Lancet* **351**: 1451–1467

© 2000 Cancer Research Campaign

Field S (1998) Breast screening issues (a study into funding, staffing, litigation and morale in the NHS Breast Screening Programme). *Royal College of Radiologists Newsletter* **54**: 12–14

Forrest AP, Stewart HJ, Everington D, et al (1996) Randomised controlled trial of conservation therapy for breast cancer: 6-year analysis of the Scottish Trial. *Lancet* **348**: 708–713

Neal AJ, Torr M, Helyer S and Yarnold JR (1995) Correlation of breast dose homogeneity with breast size using 3D CT planning and dose volume histograms. *Radiother Oncol* **34**: 210–218

Overgaard M, Hansen PS, Overgaard J, et al (1997) Postoperative radiotherapy in high risk premenopausal women with breast cancer who receive adjuvant chemotherapy. *N Engl J Med* **337**: 949–955

Parker SH, Dennis MA, Stavros AT and Johnson KK (1996) Ultrasound guided mammotomy. *Journal of Diagnosis in Medical Sonography* **12**: 113–118

SIGN (Scottish Intercollegiate Guidelines Network) (1998). Breast cancer in women: a national clinical guideline. October.

Teh W, Evans AJ and Wilson ARM (1998) [Editorial] Definitive non-surgical breast diagnosis: the role of the radiologist (editorial). *Clin Radiol* **53**: 81–84

 *© 2000 Cancer Research Campaign*

